# Lower vitamin D levels in Saudi pregnant women are associated with higher risk of developing GDM

**DOI:** 10.1186/s12884-018-1723-3

**Published:** 2018-04-10

**Authors:** Abdulrahman Al-Ajlan, Sara Al-Musharaf, Mona A. Fouda, Soundararajan Krishnaswamy, Kaiser Wani, Naji J. Aljohani, Amal Al-Serehi, Eman Sheshah, Naemah M. Alshingetti, Iqbah Z. Turkistani, A. Afrah Alharbi, Buthaynah A. Alraqebah, Aisha Mansoor Ali, Gawaher Al-Saeed, Nasser M. Al-Daghri

**Affiliations:** 1 Department of Clinical Lab Sciences, College of Applied Medical Sciences, King Saud University, Riyadh, 11451 Kingdom of Saudi Arabia; 2 Division of Metabolic and Vascular Health, University of Warwick, CV47AL, Coventry, UK; 3 Prince Mutaib Chair for Biomarkers of Osteoporosis, Department of Biochemistry, College of Science, King Saud University, PO Box, 2455, Riyadh, 11451 Saudi Arabia; 4 Obesity, Endocrine and Metabolism Center, King Fahad Medical City, Faculty of Medicine, King Saud bin Abdulaziz University for Health Sciences, Riyadh, 11525 Saudi Arabia; 5 Maternal-Fetal Medicine Department, King Fahad Medical City, Riyadh, 59406 Saudi Arabia; 6 Diabetes Care Center, King Salman Bin Abdulaziz Hospital, Riyadh, Saudi Arabia; 7 Obstetrics and Gynaecology Department, King Salman Bin Abdulaziz Hospital, Riyadh, 11564 Saudi Arabia; 8 Department of Medicine, Endocrinology Division, College of Medicine, King Saud University, Riyadh, 11461 Saudi Arabia; 9 Department of Obstetrics and Gynecology, College of Medicine, King Saud University, Riyadh, 11461 Saudi Arabia

**Keywords:** 25(OH) D, Gestational diabetes mellitus, Oral glucose tolerance test, Type 2 diabetes mellitus, Insulin resistance

## Abstract

**Background:**

Gestational diabetes mellitus (GDM) has serious consequences such as increased risks of preeclampsia, macrosomia and cesarean delivery. Even though the mechanistic basis of GDM has not been completely understood, several risk factors have been identified and one of these is vitamin D. However, the link between vitamin D deficiency and development of GDM is yet to be proven with certainty.

**Methods:**

This study aimed to investigate the link between the incidence of GDM and serum vitamin D level in pregnant women of Saudi Arabia. 515 Saudi women (ages 18–46) in their 24-28th week of pregnancy, visiting various hospitals of Riyadh, participated in this study. Serum vitamin D and various biochemical and anthropometric parameters were determined in the first trimester and the recruits were screened for GDM by OGTT according to IADPSG criteria in their 2nd trimester. The association between vitamin D deficiency and development of GDM was calculated based on odds ratio of the incidence of GDM among vitamin D deficient and normal women.

**Results:**

In this study cohort of 515 pregnant women, in the first trimester vitamin D deficiency (< 50 nmol/l) was detected in 425 (82.5%). On their 2nd visit (2nd trimester), 116 (27.7%) were diagnosed with GDM out of 419 with OGTT, according to IADPSG criteria. GDM risk was significantly higher among vitamin D deficient than non-deficient women (Odds Ratio: 2.87; Confidence Interval: 1.32–6.25; *P* = 0.008) even after adjusting for season, sun exposure and vitamin D intake (OR: 2.9; CI: 1.07–7.89). Of the various anthropometric and biochemical parameters, the GDM women differed significantly from non-GDM women with respect to serum levels of triglycerides (in mmol/l) (1.3 ± 0.6; 1.5 ± 0.6, *p* = 0.018) and fasting glucose (in mmol/l) [4.7 (4.3–5.2); 5.1 (4.6–5.6), *p* < 0.01]. Also, fasting glucose level in the 2nd trimester correlated inversely to serum vitamin D level determined during the 1st trimester (*r* = − 0.121; *p* = 0.014).

**Conclusions:**

Results of our study reveal a significantly higher risk of development of GDM among pregnant women having deficient vitamin D status.

## Background

Gestational diabetes mellitus (GDM) is increasing in prevalence in parallel with the dramatic increase in the prevalence of overweight and obesity in women of childbearing age [1]. GDM has a serious short- and long-term adverse health outcomes for both mothers and offspring. Factors which cause GDM have not been completely elucidated although several risk factors have been identified.

On the other hand, vitamin D deficiency, which was initially considered only to influence bone metabolism, is now known to exert a wide spectrum of extra-skeletal effects, including impairment of immune system [2], increased risk in cardiovascular disease and hypertension [3], disturbances in neuropsychiatric function [4] and even increased mortality [5]. The detection of vitamin D receptor expressed ubiquitously in almost all body cells suggests an involvement of vitamin D-mediated effects on critical metabolic pathways [6]. Recent epidemiological studies have found an association between 25(OH)D levels and risk of type 2 diabetes mellitus (T2DM) [7–9]. In vivo, vitamin D deficiency causes dysregulation of glucose metabolism by increasing insulin resistance through deteriorating β-cell function and mass [10, 11]. Vitamin D deficiency was also identified as a risk factor for obesity and T2DM in women at late reproductive age [12]. Furthermore, in pregnant women, vitamin D deficiency has important implications for the mother and lifelong health of the child, as it has been linked to maternal and child infections, small for gestational age (SGA), preterm delivery, preeclampsia, GDM, as well as DNA imprinting in the infant for lifelong chronic diseases [13]. Recent vitamin D supplementation (up to 4000 IU/d) studies involving pregnant women have suggested risk reduction in infection, preterm labor, and preterm birth [14]. However, observational studies about maternal vitamin D status and risk of GDM are conflicting, mostly due to differences in population characteristics such as ethnicity, geographic location, age of gestation at sampling and diagnostic criteria for GDM [15].

Vitamin D deficiency (< 50 nmol/L) which is found to be common globally and in various ethnicities, has a prevalence of 26–98% in pregnancy [16–18]. It has been reported to be high among women of child-bearing age in Saudi Arabia with approximately one third of reproductive-age Saudi women being vitamin D deficient [19] and a recent report showing < 5% having sufficient levels [20]. High rates of GDM incidence have been reported from Saudi Arabia, considered to be among the highest in the world [21].

Despite several studies, the link between GDM and maternal vitamin D status is yet to be fully understood. We aimed to investigate the effect of serum vitamin D level in the incidence of GDM along with its consequences on various biochemical parameters in pregnant women in Saudi Arabia, a country with a high prevalence of obesity, vitamin D deficiency and GDM.

## Methods

### Subjects

A total of 515 Saudi women identified to be at high risk of GDM (personal history of GDM or polycystic ovarian syndrome, glycosuria, family history of T2DM, severe obesity, macrosomia), recruited at various hospitals in Riyadh, Saudi Arabia were included in this study. In brief, only pregnant Saudi women aged 18–35 were included and non-Saudis were excluded to have a relatively homogenous cohort. Additional criteria for inclusion/exclusion have been described previously [20]. Sample size was determined using a level of confidence is at 0.05 and study power at 80% with the ratio of unexposed to exposed group 1:1 and the frequency of the outcome = 10%. The required sample is 438 pregnant women. To adjust for non-response rates, an extra 10% of the calculated sample will be added to sum up with 515 pregnant women.

Written informed consent was obtained from each subject before inclusion in the study. The study was approved by the Ethics Committee of the College of Science, King Saud University, Riyadh, Kingdom of Saudi Arabia (KSA).

### Anthropometry and blood collection

Pregnant women in their first trimester pre-natal visit were subjected to anthropometric and blood withdrawal procedures as described previously [20].

### Sample analyses

Blood samples collected from the subjects during their first visit were analyzed for 25(OH)D and various biochemical parameters. Serum 25(OH)D was determined as described before [20] with a Roche Elecsys modular analytics (Cobas e411) using an electrochemiluminescence immunoassay (Roche Diagnostics, GmbH, Mannheim, Germany) and commercially available IDS kits (IDS Ltd., Boldon Colliery, Tyne & Wear, UK). Variation for the 25(OH)D ELISA were 5.3% and 4.6%, respectively, with 100% cross-reactivity to 25(OH)D3 and 75% cross-reactivity to 25(OH)D2. It should be noted that the laboratory where samples were analyzed (Biomarkers Research Program, BRP) is a participating entity in the Vitamin D External Quality Assessment Scheme (DEQAS), and Quality Assurance (QA) standards are maintained by ISO 9000 and 17,025. The QA department audits the BRP laboratory at regular intervals. Serum glucose, lipid profile, calcium, and phosphorous were measured using a chemical analyzer (Konelab, Espoo, Finland). Serum-free insulin concentration was determined by electro-chemiluminescence method (Cobas e411; Roche Diagnostics, Mannheim, Germany).

### Oral glucose tolerance test (OGTT)

Out of 515 pregnant women recruited for the study, only 419 visited the hospitals during their 2nd trimester and consented for OGTT. Dropout rate was 19.2%. OGTT was conducted with ingestion of seventy-five gram glucose and GDM was diagnosed according to International Association for Diabetes in Pregnancy Society Group (IADPSG) guidelines [22], if one of the following applies: fasting glucose ≥5.1 mmol/l, 1 h glucose ≥10 mmol/l or 2 h glucose ≥8.5 mmol/l.

### Data analysis

Data was analyzed using the SPSS statistical Package (22.0 Version). The Kolmogorov-Smirnov test was used to analyze the normal distribution of the variables. The continuous normal variables are presented as mean ± standard deviations, while non-normal continuous variables are presented as medians and percentiles. Frequencies and percentages were used to present categorical variables. Hypothesis testing for significant differences was performed using independent Student t-test and Mann-Whitney U test for both normal and non-normal variables, respectively. Hypothesis testing for significant association was performed using chi-square test of independence. Logistic regression was performed taking GDM status as a dependent variable and vitamin D deficiency status as an independent variable. The statistical analysis was conducted at 95% confidence level and a *P*-value < 0.05 was considered statistically significant.

## Results

### Prevalence of vitamin D deficiency

Based on the recently recognized vitamin D cut-off [23], we classified pregnant women having < 50 nmol/l as deficient and those having > 50 nmol/l as non-deficient/normal. According to this criteria, out of 515 subjects of our study, 425 (82.5%) had deficient levels of vitamin D while the rest 90 (17.5%) were vitamin D sufficient. Severe vitamin D deficiency (< 25 nmol/l) was observed in 198 (38.4%) subjects.

### Prevalence of GDM

Out of the 419 subjects subjected to OGTT on the second visit, 116 (27.7%) were diagnosed as positive for GDM.

### Comparison of maternal and biochemical parameters of GDM women with non-GDM women

The non-GDM and GDM groups differed significantly with respect to serum vitamin D deficiency: 249 (82.5%) and 108 (93.1%), (*p* = 0.006); glucose (in mmol/l): [4.7 (4.3–5.2)], [5.1 (4.6–5.6)], (*p* < 0.01); age (years): (28.6 ± 5.1), (30.3 ± 5.6), (*p* = 0.03); HbA1c: (5 ± 0.4), (5.2 ± 0.5) (*p* < 0.01); triglycerides (mmol/l): (1.3 ± 0.6), (1.5 ± 0.6), (*p* = 0.018) and insulin (in uU/ml) [8.7 (4.4–19.5)], [11.5 (6.2–19.8)], (*p* = 0.048). Lastly, all obesity indices (pre-pregnancy BMI, BMI, waist size, hip size and WHR) were significantly higher among the GDM women compared to non-GDM women (all *p*-values < 0.05) (Table 1).

**Table 1:** Subject characteristics at baseline (visit 1) defined according to GDM status (visit 2)

Parameters	Non-GDM	GDM	*P*-values
N	303	116	–
Vitamin D deficiency	249 (82.5%)	108 (93.1%)	0.006
Age (years)	28.6 ± 5.1	30.3 ± 5.6	0.003
Age at menarche (years)	12.5 ± 1.5	12.7 ± 1.8	0.39
Age at 1st pregnancy (years)	24.3 ± 4.4	24.9 ± 5	0.35
Parity	2 (1.0–3.0)	2 (1.0–4.0)	0.03
Gestational age (weeks)	11.4 ± 2.6	11.4 ± 2.9	0.98
Pre-BMI (kg/m^2^)	26.4 ± 6.1	28.8 ± 6.3	0.003
BMI (kg/m^2^)	28.1 ± 6.7	31.2 ± 7.2	< 0.001
Waist (cm)	89.3 ± 12.6	94.8 ± 13.8	0.001
Hips (cm)	107.1 ± 11.9	110.9 ± 11.6	0.011
WHR	0.8 ± 0.1	0.9 ± 0.1	0.03
Systolic blood pressure (mmHg)	113.8 ± 13.6	111.7 ± 12.5	0.23
Diastolic blood pressure (mmHg)	66.9 ± 8.7	67.7 ± 10.2	0.63
Glucose (mmol/l)	4.7 (4.3–5.2)	5.1 (4.6–5.6)	< 0.001
Insulin (μU/mL)	8.7 (4.4–19.5)	11.5 (6.2–19.8)	0.05
HbA1c (%)	5 ± 0.4	5.2 ± 0.5	< 0.001
Triglycerides (mmol/l)	1.3 ± 0.6	1.5 ± 0.6	0.02
Total cholesterol (mmol/l)	4.9 ± 1.0	5.1 ± 1.0	0.06
HDL-cholesterol (mmol/l)	1.3 ± 0.3	1.3 ± 0.3	0.22
LDL-cholesterol (mmol/l)	3 ± 0.8	3.1 ± 0.9	0.10
Calcium (mmol/l)	2.2 ± 0.2	2.1 ± 0.1	0.66
Corrected calcium (mmol/l)	2.2 ± 0.2	2.2 ± 0.1	0.94
Albumin (g/l)	35.8 ± 4.2	35.6 ± 4	0.64
Alkaline phosphatase (mmol/l)	8.3 ± 3.1	9.6 ± 3.6	0.09
PTH (pg/ml) #	10.9 (2.1–13.3)	10.9 (2.3–12.2)	0.56
Creatinine # (μmol/l)	45.1 (27.4–55.8)	45.5 (24.0–60.6)	0.93
Vitamin D (nmol/l) at visit 1 #	25.5 (17.3–41.9)	24.4 (17.4–37.1)	0.49

Data presented as Mean ± SD for continuous normal variables and medians (25th percentile – 75th percentile) for continuous non-normal variables; # indicates non-normal variables; categorical variables are presented as frequency (%). Independent sample t-test and Mann-Whitney U test are used for significance testing for Gaussian and non-Gaussian variables, respectively, while chi-square test of independence was used for categorical variable(s). Serum vitamin D level < 50 nmol/l was considered as deficient. GDM was diagnosed according to IADPSG guidelines [22]

### Analysis of association between vitamin D deficiency and GDM

Analysis of vitamin D status vs GDM incidence, as given in methods, indicated significantly higher risk for GDM among women who were deficient for vitamin D [OR: 2.87 (1.32–6.25) (*p* = 0.008)] (Table 2). The risk of development of GDM associated with vitamin D deficiency was also analyzed after adjusting the data for three sets of variables, which are known to influence carbohydrate metabolism or serum vitamin D levels. The risk of GDM among women with deficient vitamin D was sustained or increased significantly after adjusting the data for: 1) season of blood sampling, sun exposure and vitamin D intake (OR: 2.90, CI: 1.07–7.89, *p* = 0.037), 2) season of blood sampling, sun exposure and vitamin D intake, BMI at visit 1, maternal age, physical activity, family history of GDM, parity, HbA1c, WHR, triglycerides (OR: 4.41, CI: 1.19–16.42, *p* = 0.027), and adjustment 1 + adjustment 2 + gestational weight gain (OR = 6.05, CI: 1.16–31.42, *p* = 0.033).

**Table 2:** Association between GDM and vitamin D deficiency in Saudi pregnant women

Vitamin D Status	Unadjusted	Adjustment 1	Adjustment 2	Adjustment 3
< 50 nmol/l	2.87 (1.32–6.25)	2.90 (1.07–7.89)	4.41 (1.19–16.42)	6.05 (1.16–31.42)
*P* value	0.008	0.04	0.03	0.03

Data presented as Odd Ratios (95% CI); Logistic regression was used taking GDM status as dependent variables and vitamin D Deficiency status as independent variable. Adjustment 1: season of blood sampling, sun exposure and vitamin D intake (IU/day). Adjustment 2: adjustment 1 + pre-pregnancy BMI, maternal age, physical activity, Family history of GDM, parity, HbA1c, WHR, triglycerides; Adjustment 3: adjustment 1 + adjustment 2 and gestational weight gain

**Fig. 1:**
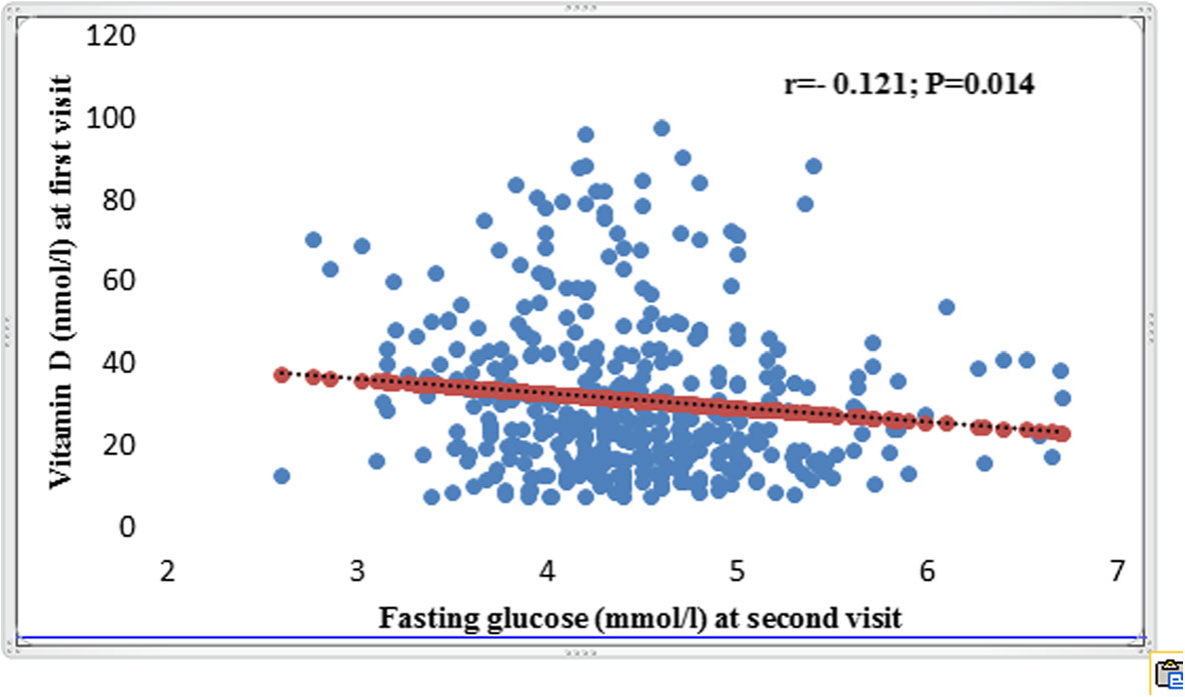
Correlational analysis of baseline serum 25(OH) D with 2nd trimester fasting glucose in pregnant women

For all the subjects (*n* = 419), serum vitamin D level measured at visit 1 was negatively correlated to fasting glucose determined at the time of 2nd visit (*r* = − 0.121; *p* = 0.014) and the results are represented as a scatter plot (Fig. 1).

## Discussion

Recent literature indicates a high level of incidence of GDM as well as vitamin D deficiency in Saudi Arabia and in this study we aimed to investigate the link, if any, between the two by determining the vitamin D level in the 1st trimester and assessing its association with GDM, determined by performing OGTT in the second semester. In a cohort of 515 pregnant women visiting different hospitals of Riyadh, Saudi Arabia, vitamin D deficiency was detected in 82.5% in the first trimester. On their 2nd visit, during the 2nd trimester of pregnancy, 27.7% of the 419 women who participated in OGTT were diagnosed with GDM. Analysis of the association between vitamin D status and incidence of GDM indicated a 2.87 fold increased risk of development of GDM among vitamin D deficient compared to vitamin D non-deficient women and this risk was sustained or substantially increased when the data was analyzed after adjusting for sunlight exposure, physical activity, calcium and vitamin D intake and other factors that are known to influence vitamin D levels. Of the various anthropometric and biochemical parameters, the GDM women differed significantly from non-GDM women with respect to serum levels of triglyceride, fasting glucose and insulin, among others. Furthermore, all the obesity indices, pre-pregnancy BMI, BMI, waist size, hip size and WHR were significantly higher in the GDM women compared to non-GDM women suggesting a strong confounder effect from obesity. Also, overall, fasting glucose levels determined in the 2nd trimester showed an inverse association with vitamin D levels determined in the 1st semester and while this association was statistically significant it was weak, which may be due to the analysis being performed at different time points. A recent Australian study involving 3393 adults (ages 18–75 years) indicated a direct, protective effect of higher 25(OH)D on fasting plasma glucose and HbA1c, two criteria for assessment of risk of T2DM. Overall, results of our study indicate a significant association between vitamin D deficiency and increased risk of GDM development during the course of pregnancy.

Vitamin D deficiency is common in healthy Saudi adults and is more pronounced in females and especially in the younger age groups [24]. In a recent study performed in Riyadh, the prevalence of vitamin D deficiency (25(OH)D < 50 nmol/l) was high in both premenopausal and postmenopausal groups (80% and 68%, respectively) during the summer, as well as during the winter (85% and 76%, respectively) [25]. Vitamin D deficiency has been reported to be common among pregnant Saudi women living in Riyadh as observed by Al-Farris, recently [26].

The prevalence of GDM varies from 1 to 20%, and is rising worldwide, and the amount of GDM varies in direct proportion to the prevalence of T2DM in a given population, or ethnic group. In a recent retrospective cohort study (2013) involving 3041 women who delivered at King Khalid University Hospital, 569 (18.7%) had GDM [21]. Another study in Saudi Arabia (2015), adopting IADPSG criteria, identified 183 out of a total of 445 women (22.1%) as having GDM [26]. In a prospective cohort study (2015) involving 277 Saudi women who underwent OGTT, 47 (16.9%) were diagnosed by the former American Diabetes Association (ADA) criteria and 115 (41.5%) by the IADPSG criteria, indicating wide difference on the rates of incidence GDM depending on the method adopted. The IADPSG criteria identified all women with GDM by the former ADA criteria and an additional 68 cases [27]. In this context, our results indicating a 30.5% GDM, adopting the IADPSG criteria, may be representative of the current levels of GDM in this region.

The influence of vitamin D on the development of GDM during the course of pregnancy appears uncertain as revealed by the analysis of several individual as well as meta-analysis reports. A meta-analysis of 20 Observational studies that comprised 9209 participants showed that women with vitamin D deficiency experienced a significantly increased risk for developing GDM with a little heterogeneity [28]. A systematic review and meta-analysis of 24 observational studies (2013) found that women with circulating 25(OH)D level < 50 nmol/l in pregnancy experienced an increased risk of preeclampsia and GDM [29]. Also, a systematic review and meta-analysis to study the link between vitamin D and gestational diabetes (2012) indicated a significant inverse relation between serum 25(OH)D and the incidence of GDM and vitamin D deficiency (25(OH)D < 50 nmol/l) in pregnancy was significantly related to the incidence of GDM [30]. However, in a prospective cohort study (Spain, 2015) involving 2382 pregnant women, overall, 31.8% and 19.7% vitamin D insufficiency [25(OH)D3 20–29.99 ng/ml] and deficiency [25(OH)D3 < 20 ng/ml], respectively, showed no association between maternal 25(OH)D3 concentration and risk of GDM [31].

A similar level of uncertainty is suggested by results available from a large number of vitamin D supplementation studies. Meta-analysis (2015) performed on 13 randomized controlled trials (RCTs) (*n* = 2299) performed to study the effect of vitamin D supplementation during pregnancy on maternal and neonatal outcomes concluded that incidence of GDM was not influenced by vitamin D supplementation [32]. This was, despite the increase in circulating 25(OH)D levels that was associated with vitamin D supplementation. However, across RCTs, the doses and types of vitamin D supplements, gestational age at first administration, and outcomes were heterogeneous. A systematic review of seventy-six studies (2014) involving vitamin D supplementation in pregnancy, sponsored by The National Institute for Health (NIH) Research, found considerable heterogeneity between the studies and for most outcomes, including GDM, there was conflicting evidence [33].

High-parity and older age have previously been found to be important risk factors for GDM in the Saudi population [34], where multiparous women were found to be > 8 times increased risk of GDM than nulliparous women. The same study also reported a 2% to 21% increase in GDM incidence when age increased from 20 to 40 years. In addition to high-parity and older age, higher maternal weight was shown to be associated with higher rates of GDM by an earlier study on Saudi women conducted in the 90s [35].

The susceptibility of Saudi women to high rates of GDM may be related to the cultural restrictions on female physical activities that may lead to physical inactivity and increased access to fast foods, both causing obesity. A recent review has shown high prevalence among Saudi women of smoking (up to 9%), hypertension (22%), diabetes (up to 27.6%), overweight (27%), and obesity (40%), and physical inactivity (53.2% to 98.1%), hypercholesterolemia (24.5%) and metabolic syndrome (MetS) (up to 40.3%) [36]. MetS is a cluster of established risk factors that together increase predisposition to major chronic diseases such as heart diseases and diabetes mellitus and a recent study (2014) on adults from several regions of Saudi Arabia confirmed > 28% incidence of MetS [37]. Vitamin D deficiency, a widely prevalent condition in Saudi Arabia, has been cited to be associated with MetS in several cross-sectional studies involving middle-aged East Asians and Europeans, but not in multicultural populations of United States and New Zealand [38]. Our results may mean that MetS is a converging point of vitamin D deficiency and obesity related issues eventually leading to increased rates of GDM in Saudi Arabian women.

A consistent association between the obesity levels, as determined from BMI and other indices, and GDM observed in this study suggests a confounding role from obesity. Altogether, results from our study performed on women living in a region which is reported to have high rates of obesity and T2DM, in general, clearly indicate a 2.8-fold increased risk among women who are vitamin D deficient.

## Conclusions

Conflicting reports currently exist on the association between maternal serum vitamin D status and GDM. Our assessment of GDM status and serum vitamin D levels in pregnant women of Saudi Arabia, a population known to suffer from high incidence of both GDM and vitamin D deficiency, suggests an increased risk of development of GDM in women who are deficient for vitamin D when compared to their non-GDM counterparts. Further studies such as a robust randomized controlled trial involving dietary supplementation of vitamin D during pregnancy may clearly define the role of vitamin D in development of GDM in Saudi women.

## Abbreviations

25(OH)D: 25 hydroxy Vitamin D
BMI: Body mass index
GDM: Gestational diabetes mellitus
IADPSG: The International Association of the Diabetes and Pregnancy Study Groups
OGTT: Oral glucose tolerance test
T2DM: Type 2 Diabetes Mellitus
WHR: Waist to height ratio
